# Reprogramming cell fate: a changing story

**DOI:** 10.3389/fcell.2014.00046

**Published:** 2014-08-27

**Authors:** Michael T. Chin

**Affiliations:** Division of Cardiology, Department of Medicine, University of WashingtonSeattle, WA, USA

**Keywords:** direct reprogramming, transdifferentiation, lineage determination, regenerative medicine, cell fate

## Abstract

Direct reprogramming of adult, lineage-determined cells from one cell fate to another has long been an elusive goal in developmental biology. Recent studies have demonstrated that forced expression of lineage-specific transcription factors in various differentiated cell types can promote the adoption of different lineages. These seminal findings have the potential to revolutionize the field of regenerative medicine by providing replacement cells for various degenerative disorders. Current reprogramming protocols, however, are inefficient in that relatively few cells in a given population can be made to undergo reprogramming and the completeness and extent of reprogramming that occurs has been questioned. At present, the fundamental molecular mechanisms involved are still being elucidated. Although the potential clinical applications are extensive, these issues will need to be addressed before direct reprogramming may be used clinically. This review will give an overview of pioneering studies in the field, will describe what is known about direct reprogramming to specific lineage types, will summarize what is known about the molecular mechanisms involved in reprogramming and will discuss challenges for the future.

## Introduction

A fundamental question in cell biology is whether the acquisition of a particular cell fate during embryonic development is reversible or changeable, and to what extent. From a practical standpoint, this question is also directly relevant to regenerative biology and its potential application to clinical medicine. For many years, the answer to this question has been a qualified affirmative, although progress has been mostly limited until the last decade. The first demonstration that somatic cell nuclei could be reprogrammed to direct enucleated oocytes to form mature fertile animals was achieved in amphibians (Gurdon et al., 1958). This technology was later used to clone mammals, nearly four decades later (Campbell et al., 1996; Wakayama et al., 1998). Although these studies demonstrated the feasibility of somatic nuclear reprogramming, the overall efficiency was low (1–2%) and worked better with nuclei from cells that were less differentiated, suggesting that epigenetic modifications are likely involved.

At the cellular level, early studies showing that 5-azacytidine treatment, which inhibits DNA methylation, could convert cultured fibroblast cell lines to myocytes, chondrocytes, and adipocytes suggested that differentiated cells could undergo transdifferentiation and that this process was under epigenetic control (Taylor and Jones, 1979). Subsequent studies on human amniocyte- mouse myocyte heterokaryons were able to demonstrate that the muscle phenotype was dominant and that cytoplasmic factors caused activation of muscle genes in the human nuclei (Blau et al., 1983). A single dominant acting bHLH transcription factor, MyoD, was later identified by its ability to transform cultured fibroblasts into myoblasts by activating muscle-specific genes (Lassar et al., 1986; Davis et al., 1987). In other terminally differentiated cell types, MyoD could activate muscle specific genes but could not suppress the starting cell phenotype, demonstrating that there are intrinsic cellular roadblocks to reprogramming (Weintraub et al., 1989). Nevertheless, this discovery prompted searches for other dominant acting transcription factors that could single handedly transform cells from one lineage to another, however, the results were largely disappointing. In general, cell fate switching seemed to occur more readily between related cell types, presumably due to similar epigenetic landscapes. Examples include conversion of primary B cells to macrophages by the transcription factor C/EBPa (Xie et al., 2004), activation of erythroid-megakaryocyte gene expression in monocytes by the transcription factor GATA1 (Visvader et al., 1992; Kulessa et al., 1995; Heyworth et al., 2002) and induction of myeloid gene expression in hematopoietic precursors by the transcription factor PU.1 (Nerlov and Graf, 1998).

## Reprogramming to pluripotency by multiple transcription factors

The advent of technologies that facilitated global transcriptional profiling in cells and tissues allowed researchers to identify large numbers of genes that are differentially expressed in different cell types. Presumably, some of the factors that were differentially expressed in different cell lineages would contribute to the maintenance of the particular cell type. This presumption led to a pioneering study in which 24 candidate transcription factors identified in embryonic stem cells were expressed simultaneously in fibroblasts to determine whether they could confer a pluripotent phenotype, and were then gradually reduced in number to the minimum necessary to induce pluripotency, resulting in the breakthrough discovery of iPS cells. In this landmark study, fibroblasts could be reprogrammed for the first time into pluripotent cells through the forced expression of four defined factors: Oct3/4, Sox2, Klf4, and c-Myc (Takahashi and Yamanaka, 2006). These cells could be injected into blastocysts and contribute to all three germ layers of the developing organism, and thus can be used to generate a variety of cell types for tissue regeneration. The generation of iPS cells and their potential for use in research and therapy has discussed in several recent review articles and will not be discussed in detail (Hanna et al., 2010; Robinton and Daley, 2012). iPS cells and embryonic stem cells can be differentiated directly to a variety of cell types through a process known as “directed differentiation” using defined factors such as bone morphogenetic proteins (BMPs), Activin, Wnts, and Fibroblast Growth Factors (FGFs). Although the generation of iPS cells represents a major advancement in stem cell biology, the process is inefficient and time consuming, which will be compounded if the derived iPS cells will then be used for directed differentiation. These factors can limit their practical use in clinical settings.

## Direct reprogramming of cell fate from one type to another

Direct reprogramming will theoretically facilitate the generation of clinically relevant cell types for organ repair from abundant, easy to obtain patient-derived cells such as fibroblasts, without the need for obtaining pluripotent stem cells. Generally this is accomplished through forced expression of lineage-specific transcription factors and has been used to promote reprogramming to a variety of cell types, such as skeletal muscle (Lassar et al., 1986; Davis et al., 1987; Weintraub et al., 1989), hepatocytes (Huang et al., 2011; Sekiya and Suzuki, 2011), neurons (Vierbuchen et al., 2010), pancreatic islet cells (Ferber et al., 2000; Zhou et al., 2008), endothelial cells (Ginsberg et al., 2012), smooth muscle cells (Cordes et al., 2009; Karamariti et al., 2013), and cardiac muscle (reviewed in Addis and Epstein, 2013). Direct reprogramming is conceptually attractive because in general it does not require reversion to a pluripotent state and represents a direct conversion from one cell lineage to another. It also provides the opportunity to directly convert cells *in situ*, which would be important in regenerative strategies. Several excellent reviews have been published recently on this subject (Vierbuchen and Wernig, 2012; Addis and Epstein, 2013; Morris and Daley, 2013). In general, reprogramming seems to work better when the starting cells share similar embryonic germ cell layer origins, but has been demonstrated to convert fibroblasts (mesoderm) to neurons (ectoderm), indicating that conversion across germ cell layers is possible (Vierbuchen et al., 2010). Although several different types of cells can undergo direct reprogramming to many different cell types (reviewed in Morris and Daley, 2013), we will focus primarily on what is known about direct reprogramming of fibroblasts, since they are generally ubiquitous, abundant and readily available for clinical use. Reports of direct fibroblast reprogramming are summarized in Table 1. We will also focus on directing cell fate conversion to neurons and cardiac myocytes, two cell types from organs that do not regenerate well, and are thus highly relevant to clinical regenerative medicine.

**Table 1:** **Reports of direct reprogramming of fibroblasts**.

**Reprogrammed cell type**	**References**
Skeletal muscle	Lassar et al., 1986; Davis et al., 1987; Weintraub et al., 1989
Hepatocytes	Huang et al., 2011; Sekiya and Suzuki, 2011
Neurons	Vierbuchen et al., 2010; Ambasudhan et al., 2011; Caiazzo et al., 2011; Pang et al., 2011; Qiang et al., 2011; Son et al., 2011; Yoo et al., 2011; Lujan et al., 2012; Liu et al., 2013
Cardiomyocytes	Ieda et al., 2010; Efe et al., 2011; Pfisterer et al., 2011; Chen et al., 2012; Inagawa et al., 2012; Islas et al., 2012; Jayawardena et al., 2012; Protze et al., 2012; Qian et al., 2012; Song et al., 2012; Addis et al., 2013; Christoforou et al., 2013; Fu et al., 2013; Hirai et al., 2013; Nam et al., 2013; Wada et al., 2013; Hirai and Kikyo, 2014; Ifkovits et al., 2014; Muraoka et al., 2014
Smooth muscle cells	Cordes et al., 2009; Karamariti et al., 2013
Macrophages	Feng et al., 2008
Pancreatic islet cells	Lumelsky, 2014
Neural precursors	Mitchell et al., 2014b; Zhu et al., 2014

## Direct reprogramming to neurons

Direct reprogramming of fibroblasts to neuron-like cells was first achieved by overexpression of a pool of 19 virally expressed candidate genes that were known to be neuron-specific, play a role in neuronal differentiation or implicated in epigenetic reprogramming (Vierbuchen et al., 2010). By systematic removal of specific candidate genes and repeated transduction, these investigators were further able to demonstrate that a minimal combination of three transcription factors, Ascl1, Brn2, and Myt1l were able to rapidly reprogram embryonic and neonatal mouse fibroblasts to neuron-like cells that expressed multiple neuron-specific proteins, demonstrated spontaneous action potentials and were able to form functional synapses. The majority appeared to be cortical, glutamatergic excitatory neurons. Subsequent studies were able to demonstrate that the combination of Ascl1, Lmx1a, and Nurr1 can convert mouse fibroblasts to dopaminergic neurons (Caiazzo et al., 2011), the combination of Ascl1, Brn2, Myt1l, Lhx2, Hb9, Isl1, and Ngn2 can convert mouse fibroblasts to motor neurons (Son et al., 2011) and that the combination of Brn2, Sox2, and Foxg2 could convert mouse fibroblasts to neuronal precursor cells (Lujan et al., 2012). Ascl1, Brn2, and Myt1l have also been shown to directly convert striatal astrocytes into neurons *in vivo* (Torper et al., 2013). NeuroD has also been shown to directly reprogram reactive glial cells into functional neurons within the cerebral cortex after brain injury (Guo et al., 2014).

Parallel studies on human fibroblasts were able to show that various combinations of factors such as Ascl1, Brn2, Myt1l, and NeuroD1 (Pang et al., 2011); Ascl1, Myt1l, NeuroD2, miR-9/9, and miR-124 (Yoo et al., 2011); or Brn2, Myt1l, and miR-124 (Ambasudhan et al., 2011) could reprogram these cells to glutamatergic neurons. A group of five factors (Ascl1, Brn2, Myt1l, Olig2, and Zic1) could also reprogram human skin fibroblasts into glutamatergic neurons and was used to generate induced neurons from patients with Alzheimer's Disease (Qiang et al., 2011). Similarly, the combination of Ascl1, Brn2, Myt1l, Lmx1a, and Foxa2 (Pfisterer et al., 2011) or the combination of Ascl1, Lmx1a, and Nurr1 (Caiazzo et al., 2011) could promote the formation of dopaminergic neurons from human fibroblasts. Human fibroblasts could also be directly reprogrammed into motor neurons by the combination of Ascl1, Brn2, Myt1l, Lhx2, Hb9, Isl1, and Ngn2 (Son et al., 2011).

## Direct reprogramming of fibroblasts to cardiomyocytes

The first demonstration that mouse fibroblasts could be directly reprogrammed to induced cardiac myocyte-like cells (iCMs) was achieved using an approach similar to that used to generate iPS cells and induced neuronal cells. A pool of 14 candidate factors was initially shown to induce cardiomyocyte-like cells and then the pool was narrowed down to the combination of Gata4, Mef2c, and Tbx5 (GMT) (Ieda et al., 2010). Only a small percentage of fibroblasts were directly reprogrammed, however, and although they had many features of cardiac myocytes, their transcriptional patterns were distinct from neonatal cardiomyocytes. In addition, only a small percentage of the cells could spontaneously contract. Another approach using a different strategy of transiently expressing the pluripotency factors Oct4, Sox2, Klf4, and c-Myc, then culturing the cells in defined media conditions commonly used in the stem cell field to promote cardiac differentiation, including the JAK inhibitor JI1, was also successful (Efe et al., 2011). Another group reported that the GMT factor combination was able to induce expression of cardiac genes, but did not produce any contracting cells (Chen et al., 2012), raising doubts about the efficacy and efficiency of the procedure. Two subsequent studies, however, were able to demonstrate that the retroviral expression of GMT transcription factors could directly reprogram fibroblasts at the site of myocardial injury and decrease infarct size, especially when given in conjunction with thymosin β 4 (Inagawa et al., 2012; Qian et al., 2012). A different group reported that direct reprogramming of mouse fibroblasts was more efficient if the transcription factor Hand2 was added in conjunction with GMT, both *in vitro* and *in vivo* after myocardial injury (Song et al., 2012). A subsequent study evaluated the effect of three factor combinations from a pool of 10 candidate factors and determined that Tbx5, Mef2c, and Myocardin induced a broader spectrum of myocardial genes than Gata4, Mef2c, and Tbx5 (Protze et al., 2012). Another study investigated the potential for microRNAs to reprogram mouse fibroblasts to cardiac myocyte like cells and determined that the combination of miR-1, miR-133, miR-208, and miR-499, in conjunction with JAK inhibitor I was sufficient both *in vitro* and *in vivo* (Jayawardena et al., 2012). Others have tried to optimize the reprogramming further and have found that addition of Myocardin, SRF, Mesp1, and Smarcd2 to Gata4, Mef2c, and Tbx5 can enhance the process (Christoforou et al., 2013). To improve the likelihood of obtaining functional cardiac myocytes, another group used fibroblasts containing a calcium sensitive GFP reporter and found that the combination of Hand2, Nkx2-5, Gata4, Mef2c, and Tbx5 could reprogram adult mouse fibroblasts 50 fold more efficiently than GMT alone and that the induced cardiac myocytes demonstrated robust calcium oscillations and spontaneous beating (Addis et al., 2013). The efficiency of conversion by GMT to spontaneously contracting cardiomyocyte-like cells was also reportedly improved by the tethering of the MyoD activation domain to each of these transcription factors (Hirai et al., 2013). A follow up study showed that direct reprogramming with these factors was further enhanced by inhibition of repressive histone modifications (Hirai and Kikyo, 2014).

Direct reprogramming of human fibroblasts to cardiac myocyte-like cells has also been reported, but with different factor requirements. Forced expression of the transcription factors Ets2 and Mesp1 or recombinant ETS2 and MESP1 proteins modified with cell penetrating peptides were sufficient to convert human neonatal foreskin fibroblasts into cardiac progenitors (Islas et al., 2012). The transcription factors Gata4, Hand2, myocardin, and Tbx5 in conjunction with microRNAs miR-1 and miR-133 were sufficient to directly reprogram neonatal foreskin, adult cardiac and adult dermal fibroblasts to cardiomyocyte-like cells (Nam et al., 2013). The function of miR-133 in this context is reportedly to suppress Snai1 and fibroblast genes (Muraoka et al., 2014). The addition of Myocardin and Mesp1 to GMT was reported to reprogram human cardiac fibroblasts to cardiomyocyte-like cells that express a broad array of cardiac genes and exhibit calcium oscillations (Wada et al., 2013). GMT factors in conjunction with MESP1 and ESRRG have also been reported to directly reprogram several types of human fibroblasts to cardiomyocyte-like cells (Fu et al., 2013).

These studies in aggregate demonstrate that multiple transcription factors and microRNAs can contribute to direct reprogramming of fibroblasts. One potential contributor to the variation between these studies is the lack of consensus criteria for assessing the degree of reprogramming. The development and use of standardized criteria for evaluation of transdifferentiation to iCMs, in terms of gene expression, structural, and functional characteristics has been suggested for these types of experiments (Addis and Epstein, 2013).

## Mechanisms of direct reprogramming

The mechanisms of direct reprogramming are incompletely understood. While it is well established that transcription factors drive the process and that microRNAs can contribute, it is less clear how cells maintain lineage and in general prevent the development of inappropriate cell types. The process involves activation of target genes, which usually occurs within hours to days (Ieda et al., 2010; Vierbuchen et al., 2010), direct transition from one state to another, without the need to go through a pluripotent state (Zhou et al., 2008; Ieda et al., 2010), does not require cell division, in contrast to induction of pluripotency (Zhou et al., 2008; Hanna et al., 2009; Heinrich et al., 2010; Vierbuchen et al., 2010) and is stable after removal of reprogramming factors (Zhou et al., 2008; Huang et al., 2011; Sekiya and Suzuki, 2011). The interactions between the positive actions of transcription factors and the negative influences of chromatin architecture and epigenetic modifications are currently under investigation. It has long been known that the genome encodes many binding sites for a given transcription factor, but the local chromatin structure only allows certain sites to be accessible, in a cell type-specific fashion. An example is the hematopoietic transcription factor Scl/Tal, which binds to different sites in different hematopoietic cell types (Wilson et al., 2010; Palii et al., 2011). Unneeded areas of the genome are packaged into heterochromatin and are generally not accessible to transcription factors (Beisel and Paro, 2011). To achieve reprogramming, not only must the reprogramming factors find appropriate binding sites, they must also remodel chromatin appropriately to allow ancillary factors to bind and activate a cell type-specific program. This challenge may explain the general requirement during direct reprogramming for multiple transcription factors that act cooperatively to remodel diverse areas throughout the genome. Another hypothesis being considered is that the reprogramming factors act as “pioneer” transcription factors that can bind to their cognate sites regardless of chromatin configuration (Zaret and Carroll, 2011). In this model, the pioneering factors can bind to their cognate sites and displace nucleosomes, thereby creating a permissive environment for other factors to bind. Given that some cell types are not amenable to direct reprogramming and that related cells are generally more amenable to reprogramming, it is likely that some degree of initial chromatin accessibility or “open access” is necessary even for factors that have “pioneer” capability. Studies on the muscle specific factor MyoD demonstrate that cells susceptible to reprogramming have accessible enhancer elements that allow MyoD binding despite being in an overall repressive state where gene transcription is turned off. Ectopic MyoD was able to quickly bind the enhancer element in the first 24 h, followed by acquisition of H3K4me marks by 48 h (Taberlay et al., 2011).

Direct reprogramming to different cell types occurs at varying efficiency but is usually low. In addition, successful reprogramming often requires high expression levels of reprogramming factors. Accordingly, another postulated mechanism of reprogramming involves transient accessibility to transcription factor binding sites during nucleosome turnover or other mechanisms in which DNA becomes accessible in a stochastic fashion, such as during different phases of the cell cycle (Egli et al., 2008; Vierbuchen and Wernig, 2012).

## Current limitations and challenges for the future

In addition to low efficiency, another major limitation of direct reprogramming as a strategy to regenerate tissues is the presence of epigenetic memory. Epigenetic memory specific to the original cell type has been well documented in iPS cells (Kim et al., 2010, 2011; Polo et al., 2010). Despite induction of gene expression consistent with reprogramming to another cell type, in multiple cases, some residual gene expression specific to the cell type of origin persists (Feng et al., 2008; Marro et al., 2011). Induced neurons derived from hepatocytes still demonstrate some hepatocyte-specific gene expression (Marro et al., 2011), while induced macrophages derived from fibroblasts still express some fibroblast genes (Feng et al., 2008). In many reported cases of direct reprogramming, only a small set of target genes were assessed, and in cases where more thorough transcriptomic analysis has been performed, there is significant divergence in gene expression patterns from native cells (Ieda et al., 2010; Sekiya and Suzuki, 2011). Since epigenetic memory has also been shown to persist in embryos generated from somatic cell nuclei (Ng and Gurdon, 2005), this problem may be challenging to resolve.

A promising alternative approach has been to use pluripotency factors in the early stage of direct reprogramming followed by induction with cell-type specific factors to promote the differentiation of fibroblasts to cardiac myocytes (Efe et al., 2011). This method is thought to induce a transient state of plasticity more amenable to direct reprogramming without full induction of pluripotency, and reportedly is much more efficient than direct reprogramming. Oct4 in particular has been implicated to play an important role in this regard (Mitchell et al., 2014a,b). To date, however, this approach is limited by persistence of pluripotency markers in the reprogrammed cells and the resulting cells have properties of atrial cardiac myocytes, which may be less useful for regenerative purposes. In general, the phenotype of directly reprogrammed cells is often immature compared to fully differentiated native cells within the target organ of interest, and this may limit their utility in regenerative medicine. For cardiac cells in particular, incomplete differentiation may prevent proper electrical and mechanical coupling, leading to arrhythmias and possibly heart failure. Strategies to promote a state of differentiation comparable to that of target tissue will also be critical to facilitate the use of these cells in regenerative medicine. Exogenous, chemically defined components such as ascorbic acid, recombinant human albumin and other small molecules may be useful in this regard (Crescini et al., 2013; Burridge et al., 2014). The utility of small molecules and chemically defined conditions in promoting direct reprogramming is well established (Lin et al., 2013; Liu et al., 2013; Ifkovits et al., 2014; Lumelsky, 2014; Zhu et al., 2014).

Overall, the potential applications of direct reprogramming to regenerative medicine are extensive. More studies are needed, however, to characterize more fully the phenotype of reprogrammed cells, particularly the extent of epigenetic memory, residual gene expression specific to the original cell type and ability to achieve an appropriate differentiation state and function similarly to native cells. Further refinement of transcription factor combinations, the use of adjunct agents that promote chromatin accessibility, the use of small molecules and the potential utility of pluripotency factors are only a few of the possible approaches to enhance direct reprogramming that are expected to evolve in the future.

### Conflict of interest statement

The author declares that the research was conducted in the absence of any commercial or financial relationships that could be construed as a potential conflict of interest.
